# Overcoming the Challenges of Intravenous Immunoglobulin Resistance in Kawasaki Disease: A Case Report

**DOI:** 10.31729/jnma.8837

**Published:** 2024-12-31

**Authors:** Rahul Kumar Chaudhary, Sajjad Ahmed Khan, Raman Kumar Sah, Shreya

**Affiliations:** 1 Department of Anesthesia, Birat Medical College Teaching Hospital, Tankisinuwari, Morang, Nepal; 2 Birat Medical College Teaching Hospital, Tankisinuwari, Morang, Nepal

**Keywords:** *case report*, *culture positive*, *IVIG resistant*, *Kawasaki disease*, *vasculitis*

## Abstract

Kawasaki disease, extremely rare vasculitis of unknown etiology characterized by inflammation of small to medium sized arteries of multiple systems. This case report enhances the knowledge and management skills of rare clinical conditions. Here we report a case of a 3-month-old infant presented with acute onset fever, rash, non-exudative conjunctivitis and swellings of hands and legs with blood culture positive sepsis and right coronary artery dilatation. The child didn't respond to treatment with the first dose of intravenous immunoglobulin. Therefore, the immunoglobulin resistant Kawasaki disease in infants needs further evaluation and clinical management at tertiary level hospitals.

## INTRODUCTION

Kawasaki disease (KD) is an acute vasculitis affecting primarily children under five years of age, with a significant risk of coronary artery aneurysms if untreated. Its etiology remains unclear, though an infectious trigger has been suggested. In developing countries, such as Nepal, KD is often underdiagnosed due to a lack of awareness and access to adequate healthcare resources, despite the availability of effective treatments like intravenous immunoglobulin (IVIG) and aspirin.^1,2^ This case highlights the importance of clinical vigilance and the implementation of established diagnostic criteria in atypical presentations. The child presented with classical features, including high-grade fever, maculopapular rash, conjunctivitis, and cervical lymphadenopathy, consistent with KD.^3^ Despite initial IVIG resistance, timely recognition and treatment resulted in a favorable outcome. However, the absence of genetic testing capabilities limited our diagnostic approach and understanding of the disease in this case.^4^

## CASE REPORT

**Figure 1:**
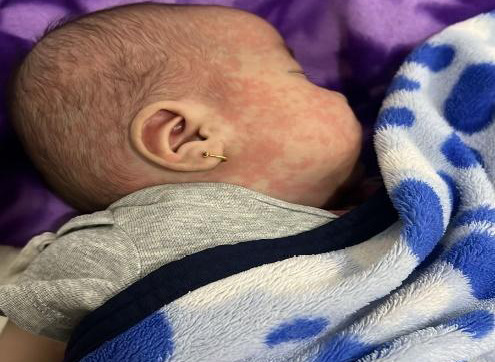
Maculopapular rashes over face of a child with Immunoglobulin resistant Kawasaki Disease.

**Figure 2:**
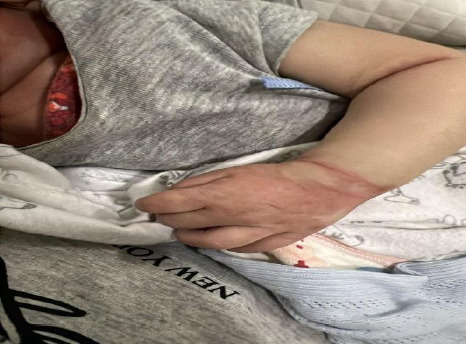
Maculopapular rashes and swelling over dorsum of hand of a child with Immunoglobulin resistant Kawasaki Disease.

**Figure 3:**
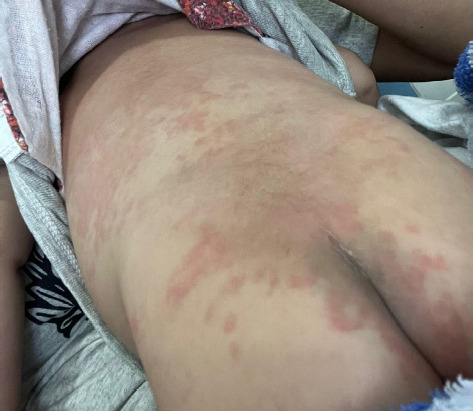
Perianal desquamation of a child with Immunoglobulin resistant Kawasaki Disease.

A 3-month-old male presented to the emergency department of our tertiary specialist unit on 13^th^ February, 2024 with the history of acute onset high grade fever (maximum 103°F) for 7 days. He had developed a maculopapular rash on hand, face and trunk (Figure 1). He also had bilateral nonexudative bulbar conjunctivitis and swelling of both hands (Figure 2) and legs on the fourth day of fever. No significant family history without any relevant past interventions. On examination, there was generalized polymorphous erythematous maculopapular rash with strawberry tongue, congestion of faucial pillars, uvula, and bilateral non-exudative conjunctivitis and, desquamation over peri-anal region (Figure 3).

His respiratory rate was 40/minute, heart rate 112/minute, blood pressure 90/50 mmHg with temperature 101.3°F and oxygen saturation was 98% in room air. Investigations showed Hemoglobin 9 gm/dl, RBC count 2.95 million/cmm, total leucocyte count 16600/cu mm. C-Reactive protein value 120 mg/l, and Erythrocyte sedimentation rate value 57 mm/hr, Ultrasonography of neck showed left cervical lymphadenopathy at level IIA measuring about 14.3 × 5.7 mm, echocardiography findings were RCA 3 mm, SD + 5.38 with normal biventricular function, enterococcus species isolated in blood culture. Liver enzymes and renal function tests were normal. This diagnosis was made as per Japanese worker's criteria. Treatment was started with intravenous ceftriaxone and on the third day of admission intravenous immunoglobulin(2gm/kg) single infusion was administered over 10 hours with aspirin 100 mg/kg/day divided every 6 hours orally for 48 hours. Even after administration of the initial dose of IVIG fever persisted for more than 36 hours hence, the diagnosis of IVIG resistant Kawasaki disease was made. So, a second dose of IVIG was administered (2gm/kg) and the child responded well with timely treatment and on 10^th^ day of admission the child was discharged and advised to continue aspirin 75mg half tablet once daily and follow up in 10 days or SOS then 3 monthly for 2 years and planned echocardiography after 3 months. There were no adverse and unanticipated events during the course of management.

## DISCUSSION

Kawasaki disease is an acute febrile mucocutaneous lymph node syndrome and is the second most common cause of vasculitis in children after Henoch Schonlein purpura.

It has replaced acute rheumatic fever as the leading cause of acquired heart disease but vast majority of patients still continue to remain undiagnosed.^5^ Early diagnosis and treatment of kawasaki disease is of utmost importance because of dreadful complications during acute illness which include myocarditis, pericarditis, valvular heart disease and coronary arteritis. Studies have shown that coronary aneurysm can occur in 20% if left untreated.^6^ It is important to remember that the diagnosis of Kawasaki disease is based entirely on the recognition of a temporal sequence of characteristic clinical findings and there is no specific laboratory test . One of the Kawasaki disease diagnostic guidelines (Japanese Ministry of Health Criteria) includes fever lasting for at least 5 days along with presence of at least 4 of the following 5 signs— (1) bilateral conjunctival injection, (2) generally non purulent, changes in mucosa of oropharynx, including oropharynx, strawberry tongue, (3) changes of peripheral extremities such as edema and/or erythema of hands or feet in acute phase, (4) rash, primarily truncal, polymorphous but non vesicular, Illness is not explained by other known disease process.^7^

Treatment with 2 gm/kg of IVIG as a single infusion, usually administered over 10-12 hours within 10 days of disease onset and ideally as soon as possible after the diagnosis and moderate to high dose aspirin should be administered until the patient become afebrile then lowered to antiplatelet doses.^8^ The IVIG resistant Kawasaki disease occurs in approximately 15% of patients and is defined by persistent or recrudescent fever 36 hours after completion of the initial IVIG infusion. Therapeutic options for the child with IVIG resistant Kawasaki disease include 2^nd^ dose of IVIG, corticosteroids and/or infliximab.^9^ Prevalence of coronary disease, 20% in children treated with aspirin alone is <5%.

Simple rash to look skin rash considering communicable entities in our clinical setup, however it will be better to investigate and identify rare immunological conditions in the era of evidence-based medicine to practice precision medicine. In patients' perspective, the symptoms were like viral illness. They started to get treatment from a nearby clinic later they came to know about the rare Kawasaki disease and treatment modality and the treatment plan was quite expensive as they required 2 doses of IVIG.

This is a rare disease and usually missed in developing countries like Nepal despite its adequate known effective clinical intervention. Our team of clinicians identifies the rare entity and efficiently manages it are the main strength of this report.

Despite such strength we were limited not to have genetic testing in our facility.
